# Identifying Strategies for Home Management of Ostomy Care: Content Analysis of YouTube

**DOI:** 10.2196/66634

**Published:** 2025-03-06

**Authors:** Marketa Haughey, David M Neyens, Casey S Hopkins, Christofer Gonzaga, Melinda Harman

**Affiliations:** 1 Department of Bioengineering Clemson University Clemson, SC United States; 2 Department of Industrial Engineering Clemson University Clemson, SC United States; 3 School of Nursing Clemson University Clemson, SC United States

**Keywords:** medical device usability, digital health, online support groups, living with chronic medical conditions, ostomy self-care, YouTube, patient education, user needs assessment, users experience, social media, ostomates, colostomy, ileostomy, usability, usefulness, utility, wearable device, medical device, support group, socials, social network, ostomy, digital, digital technology, digital intervention

## Abstract

**Background:**

The social media platform YouTube is a recognized educational resource for health information, but few studies have explored its value for conveying the lived experience of individuals managing chronic health conditions and end users’ interactions with medical device technology. Our study explores self-care strategies and end user needs of people living with a stoma because patient education and engagement in ostomy self-care are essential for avoiding ostomy-related complications. Ostomy surgery creates a stoma (an opening) in the abdomen to alter the route of excreta from digestive and urinary organs into a detachable external pouching system. After hospital discharge, people who have undergone ostomies perform critical self-care tasks including frequent ostomy appliance changes and stomal and peristomal skin maintenance.

**Objective:**

The purpose of this study was to systematically assess YouTube videos narrated by people who have undergone ostomies about their ostomy self-care in home (nonhospital) settings with a focus on identifying end user needs and different strategies used by people who have undergone ostomies during critical self-care tasks.

**Methods:**

Using predefined search terms and clear inclusion and exclusion criteria, we identified YouTube videos depicting narrators who have undergone ostomies and their ostomy self-care in home settings. Using a consensus coding approach among 3 independent reviewers, all videos were analyzed to collect metadata, data of narrators who have undergone ostomies, and specific content data.

**Results:**

There were 65 user-generated YouTube videos that met the inclusion and exclusion criteria. These videos were posted by 28 unique content creators representing a broad range of ages who used a variety of supplies. The common challenges discussed were peristomal skin complications, inadequate appliance adhesion and subsequent leakage, and supplies-related challenges. Narrators who have undergone ostomies discussed various expert tricks and tips to successfully combat these challenges.

**Conclusions:**

This study used a novel approach to gain insights about end user interactions with medical devices while performing ostomy self-care, which are difficult to gain using traditional behavioral techniques. The analysis revealed that people who have undergone ostomies are willing to share their personal experience with ostomy self-care on the web and that these videos are viewed by the public. User-generated videos demonstrated a variety of supplies used, end user needs, and different strategies for performing ostomy self-care. Future research should examine how these findings connect to YouTube ostomy self-care content generated by health care professionals and organizations and to guidelines for ostomy self-care.

## Introduction

### YouTube as Health Information Resource

Individuals are increasingly seeking health-related information on the web, and in some cases, patients rely on the internet as much or more than their physicians [1,2]. Patients have also reported using web-based resources to make health-related decisions and to manage chronic conditions [2,3].

YouTube, the largest on the web video-sharing platform worldwide for streaming a variety of user-generated content with 2.5 billion users worldwide [4] is a recognized educational resource for sharing and disseminating health-related information and influencing individuals’ viewpoints related to health care topics such as disease prevention, treatment therapy, or immunizations [2,3]. Several studies highlight the informational flaws and biases in a wide variety of YouTube health information videos related to clinical decision-making, diagnosing, treatment recommendation, and health promotion. The analysis in these studies generally focuses on the health information provided rather than the individual user experience with the health issue [2,5-9].

Studies involving so-called “e-patients” who are motivated to share health-related personal experiences on the web reveal that sharing their lived experience and knowledge of their disease can empower and engage others while also providing (and gaining) social support [10,11]. While many health-related government organizations such as the World Health Organization, the National Institute of Health, the Center for Disease Control and Prevention, and the American Red Cross use YouTube to disseminate health information [1], user-generated videos are also used as an educational resource. This suggests that user-generated content decreases the gap between health information delivered by health care professionals and the lived experience of dealing with a disease.

### Medical Device Usability in the Home Setting

Usability studies examining the interaction between medical device technology and end users are essential for developing medical devices that are safe and effective [12]. These studies allow for actively including end users in the design process while considering end user competencies and environment, thus, continuously improving medical device design to fit user needs [13]. Capturing the real world, end user experience of people with chronic conditions and their interaction with complex medical device technology in home (nonhospital) settings is challenging [12], such as having a stoma and routinely using an ostomy appliance, and to our knowledge, these studies are limited [14].

### Ostomy Surgery

**Figure 1:**
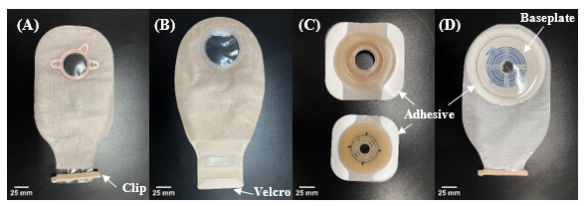
An ostomy appliance consists of an external pouching system that is attached to the abdomen of people who have undergone an ostomy using adhesive. There are various design features, including a (A) 2-piece pouch with clip closure, (B) 2-piece pouch with Velcro closure, (C) precut (top) and moldable (bottom) baseplate that is subsequently attached to the 2-piece pouch [in (A) or (B)] at the ring, and (D) 1-piece appliance with clip closure (pouch and baseplate being 1 part).

There are about one million people living with ostomy in the United States and more than 150,000 Americans undergo ostomy surgery each year [15,16]. Ostomy surgery alters the route of excreta from internal digestive and urinary organs through a stoma in the abdomen and into a detachable external pouching system (Figure 1) that fits snugly around the stoma [16]. People who have undergone ostomies perform critical self-care tasks that include frequent changing of the pouching system (3-10 changes per week) and maintaining healthy stoma and peristomal skin [17]. Therefore, there is a need to understand the usability of medical device technology used by people who have undergone ostomies in ostomy appliance change procedures and self-care.

### Ostomy Care Patient Education and Self-Management

It is widely recognized that patient education and engagement in ostomy self-care are essential for avoiding ostomy-related complications [18-20]. However, the education of new people who have undergone ostomies to gain the knowledge and skills needed for managing chronic conditions and performing self-care tasks is not well defined [18]. Ideally, according to the Wound, Ostomy, and Continence Nurses Society Clinical Guidelines and recommendations [21], new people who have undergone ostomies should obtain education from ostomy nurse specialists. Nevertheless, there is evidence of insufficient pre- and postoperative education provided to new people who have undergone ostomies related to stoma and peristomal skin care and complications [22-25]. Due to the short hospitalization trends and the limited availability of ostomy nurse specialists, there is limited teaching time and formal follow-up with the specialists [22-25]. Further, new people who have undergone ostomies may not be mentally and physically prepared for self-management of their ostomy, which requires gaining new knowledge, skills, and attitudes [26,27]. Most people who have undergone ostomies experience some long-term challenges concerning the management of daily self-care of their stoma that broadly impact their health-related quality of life and outcomes [28,29]. Peristomal skin complications are a common problem following ostomy surgery affecting over one-third of patients with ostomies within 90 days post surgery and up to 80% within 2 years [30-33]. While it is known that skin problems interfere with pouch adhesion, causing challenges with pouch leakage, and thus odor, and the necessity for frequent and unscheduled appliance changes [29], it is very difficult to study real-world responses to such challenges.

### Study Objective

Given the complexity of ostomy appliance change procedure medical technology and self-care, there is a need to assess the usability of this technology and how self-management skills are developed by people who have undergone ostomies over time. We completed a systematic analysis of user-generated videos related to ostomy self-care published on YouTube and created by individuals with expertise in managing chronic conditions about ostomy self-care in home (eg, nonhospital) settings. Our objective was to identify end user experiences with ostomy medical device technology and the different strategies and procedures used by these experts to change their ostomy appliance and perform stomal and peristomal skin care.

## Methods

### Ethical Considerations

This study was excluded from an institutional ethics board review because it assessed only copyrighted, public source information and no individual user data was included in the manuscript.

### Search Terms and Search Strategy

Search terms related to ostomy self-care were defined by the research team (Table 1) for identifying videos using the search tools within YouTube. During December 2023, YouTube was searched for relevant videos using search terms and Boolean operators under the search category “videos only” tab or “video” filter. All searches were conducted using an incognito or private browser that did not retain cookies to prevent suggested related videos from appearing on search results and to ensure that the search for each term could be replicated. The first 20 videos for each search term were reviewed and documented in a spreadsheet. No videos were included in the analysis if they appeared in the YouTube search result under the “people also watched,” “shorts,” or “sponsored websites” section as these videos may or may not have met the search term criteria. The initial search resulted in 960 videos.

**Table 1:** Search terms used to search for relevant user-generated videos published on YouTube.

Search term category	Search terms
Ostomy pouch change procedure	^a^ + (pouch OR bag) + (care OR change OR procedure)
Ostomy tips and tricks	^a^ + (pouch OR bag) + (pros and cons OR tips OR tips and tricks OR empty OR cleaning OR care OR leakage OR leakage prevention)
Ostomy self-care and home management	^a^ + (pouch change OR bag change) + (home OR self-care OR care)
Ostomy skincare	^a^ + (skin OR peristomal skin) + (care OR treatment OR irritation OR dermatitis)

^a^Is ostomy OR urostomy OR colostomy OR ileostomy OR stoma OR colorectal cancer OR Crohn disease OR inflammatory bowel disease OR IBD OR diverticulitis OR bladder cancer OR cystectomy.

### Inclusion and Exclusion Criteria

#### Overview

**Figure 2:**
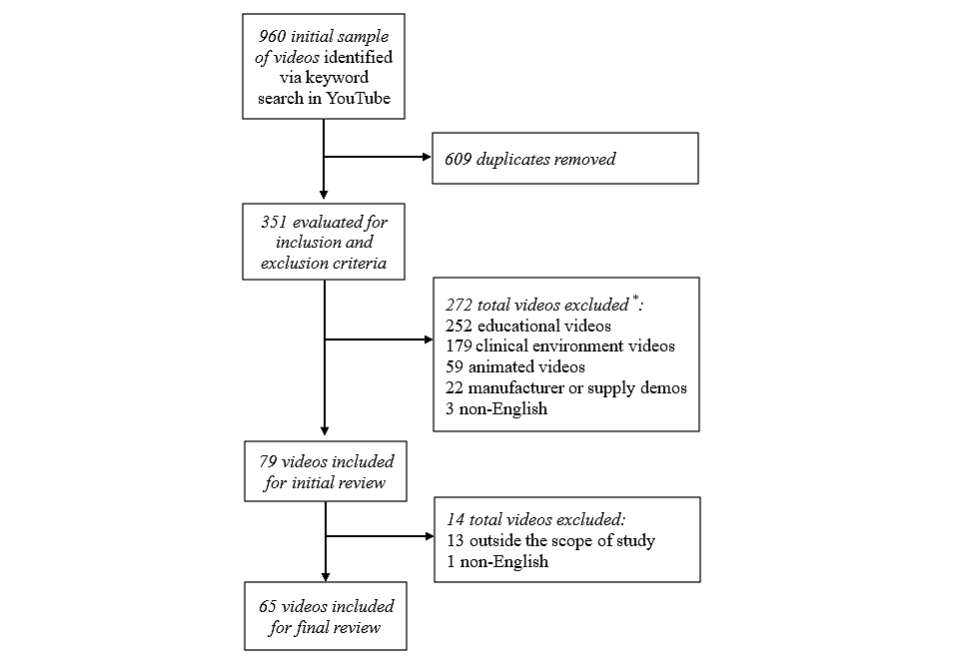
Video search inclusion and exclusion process. *Some videos were excluded for multiple criteria.

From the initial set of 960 videos, a final set of 65 videos (Figure 2) was identified for review after the removal of duplicates and the application of inclusion and exclusion criteria (Textbox 1). Duplicate videos (n=609) were identified and removed by channel grouping and comparing URLs and video titles. The inclusion and exclusion criteria were applied to eliminate educational videos (n=252), videos recorded in a clinical environment (n=179), animated videos (n=59), supply or manufacturer demonstration videos (n=22), videos in languages other than English (n=4) and those videos that were outside the scope of this study (n=13) such as broad discussions of clinical issues or ostomy support. Educational videos were defined as videos not discussing personal experiences related to ostomy self-care, for example, videos providing information related to ostomy belts.

**Specific inclusion and exclusion criteria.**

**Included videos**

- Were found using search terms
- Included a user narrative
- Involved any channel, age, gender, race, ethnicity, year, or length
- Depicted ostomy appliance change procedures in a nonhospital setting
- Discussed ostomy self-care, tips, tricks, or challenges
- Involved a person living with a stoma as a narrator
- Were published in the English language

**Excluded videos**

- Were recorded in a clinical environment or were narrated by a medical professional (not having a stoma)
- Included experiences of users who have not undergone ostomies
- Were educational videos
- Were supply or manufacturer demonstration videos
- Were animated
- Duplicated videos from other search terms
- Were published in the non-English language
- Categorized as video “shorts”
- Were outside the scope of this study

#### Data Coding

In total, 3 categories of video-specific variables were recorded**,** including video metadata, narrator data, and specific content data (Table 2). The researchers used consensus coding of the included videos, which is a well-accepted method to enhance credibility and reliability in qualitative research [34]. Before video reviews, 3 general categories and 15 variables were named and defined (Table 2). Each video was then coded individually by 2 reviewers using the defined categories and any discrepancies between reviewers were discussed. If consensus was not reached, a third reviewer reviewed the video and data coding to address bias and reach consensus and to ensure accuracy in data coding. The category definitions were then revised as needed before a final consensus coding of all the included videos by 2 reviewers.

**Table 2:** Data coding for 3 categories of videos including specific variables, and their definition and type.

Category and variable		Definition
**Video metadata**		
	Title	The title of the video by the content creator
	URL	The web address of the video
	Channel	The name of the page where videos are posted for viewing
	Posted year	The year that video was publicly posted to the YouTube platform
	Video length	The time duration of the video
	View counts	The number of times the video was viewed as of January 4, 2024
	Public ratings	The number of likes or dislikes as of January 4, 2024
**Data of narrators who have undergone ostomies**		
	Presenting assumed gender	The assumed gender of the content creator based on gender markers such as physical build, voice, clothes, and hair
	Presenting assumed age group	The assumed age group of the content creator mainly based on physical appearance and hair coloration
**Specific video content data**		
	Supplies used	Any item, product, equipment, or accessory used or discussed as used by a narrator who has undergone an ostomy during the ostomy appliance change procedure
	Appliance change frequency^a^	How frequently the narrator who has undergone an ostomy completed an appliance change process
	Type of ostomy^a,b^	The type of ostomy disclosed by the narrator who has undergone an ostomy
	Underlying condition for ostomy^a,b^	The medical condition experienced by the narrator who has undergone an ostomy and contributing to the ostomy surgery
	Common challenges^a^	Description of the problems related to ostomy, ostomy appliance change procedures, and ostomy self-care
	Expert tips and tricks	Advice or recommendation given by a narrator who has undergone an ostomy to the audience to address common challenges, aid in expediting or easing the appliance change process, or provide additional information to improve the overall user experience

^a^Variable was not communicated in every video.

^b^If not communicated in the video, the intro page and the about me page for the creator were reviewed.

## Results

### Video Metadata

After removing duplicate videos and applying the inclusion and exclusion criteria, there were 65 videos that explored different topics related to ostomy, ostomy appliance change procedures, and the self-care of people who have undergone ostomies. Included videos were posted between 2011 and 2023 and averaged about 9.5 minutes in length, over a quarter-million views, and more than 3000 likes (Table 3).

**Table 3:** The video metadata descriptive statistics for the videos included in the analysis.

Video metadata	Data
Posted year, n/N (%)	Between 2011 and 2023 (11/65, 17% and 12/65, 18% videos in 2020 and 2023, respectively)
Video length, mean (SD; range)	9:36 min (SD 6:30 s; 1:00-31:40 min)
View counts, mean (SD; range)	269,076 (SD 922,352; 1900-5,493,702) views
Public ratings, mean (SD; range)	3015 (SD 8761; 0-58,000) likes

### Data of Narrators Who Have Undergone Ostomies

There were 28 unique content creators with several individual creators posting multiple videos. Further, 2 high volume narrators who have undergone ostomies uploaded almost half (31/65) of all the videos (24 and 7 videos from the 2 most frequent content creators). Most videos included a female narrator and a narrator in the 20-30 years age range followed by a middle-aged narrator, an older narrator, and a teenage narrator (Table 4). None of the narrators who have undergone ostomies communicated their gender or age in the videos. Therefore, the presenting assumed gender was determined by the reviewers based on the gender markers such as physical build, voice, clothes, and hair and the presenting assumed age based on the physical appearance and hair coloration.

**Table 4:** Data of narrators who have undergone ostomies for videos included in the final review.

Unique narrators who have undergone ostomies (n=28)		Data, n/N (%)
**Presenting assumed gende**r		
	Female	20/28 (71% narrators who have undergone ostomies)
	Male	8/28 (29% narrators who have undergone ostomies)
**Presenting assumed age group**		
	Infant^a^	1/65 (2% videos)
	Teenager	4/65 (6% videos)
	Aged 20-30 years	42/65 (65% videos)
	Middle age	13/65 (20% videos)
	Older	5/65 (8% videos)

^a^One video was presented about an infant with an ostomy that was narrated by the parent caregiver.

### Specific Video Content Data

#### Ostomy Video Content

From the final set of 65 videos, 26/65 (40%) videos discussed ostomy self-care tips and tricks that focused most frequently on maintaining peristomal skin health, preventing leaks, pancaking, supply use, and general tips; 21/65 (32%) videos captured a full ostomy appliance change procedure and 14/65 (22%) videos included part of an ostomy appliance change procedure such as emptying, specific supply use, and skin preparation and appliance application, among others; and 4/65 (6%) videos were about the personal experience of a person living with a stoma (Table 5).

**Table 5:** Types of ostomy-related video content, its frequency, and topics discussed.

Video content and topic discussed		Video count, n		Video frequency, n/N (%)
**Tips and tricks**				26/65 (40%)
	Peristomal skin health	6		
	Leaks	5		
	Pancaking	3		
	Emptying or not emptying	3		
	Specific supply use	2		
	General	2		
	Odor	1		
	Intimacy	1		
	Adhesion	1		
	Pouch decoration	1		
**Full ostomy appliance change**				
	Ostomy appliance change procedure	21	21/65 (32%)	
**Part of ostomy appliance change**				14/65 (22%)
	Empty an appliance	5		
	Specific supply use	3		
	Skin preparation and appliance application	2		
	Change appliance after shower	1		
	Remove appliance and clean peristomal skin	1		
	Cut or fit ostomy baseplate	1		
	Appliance change omitting details	1		
**Experience**				4/65 (6%)
	Living with stoma	2		
	Living in London	1		
	Stories of people who have undergone ostomies	1		

#### Supplies Used

The narrators who have undergone ostomies did not all use identical supplies and there was a variety of types, numbers, and frequencies of supplies used during the ostomy appliance change procedures (Table 6). Among the 35 videos that captured a full or partial ostomy appliance change procedure, 25/35 (71%) included a 2-piece pouching system, and 9/35 (26%) included a 1-piece pouching system (Table 6). In 1/35 (3%) videos, the type of ostomy appliance could not be determined. Furthermore, the baseplate was cut to fit or precut by the supplier in 25/35 (71%) videos and molded by hand in 3/35 (9%) videos. In 7/35 (20%) videos, the method for sizing the baseplate by cutting or molding could not be determined.

Further, supplies used to clean the peristomal skin and remove any remaining adhesive or output included paper towel or toilet paper in 19/35 (54%) videos, adhesive remover wipes in 17/35 (49%) videos, adhesive remover spray in 14/35 (40%) videos, dry wipes in 11/35 (31%) videos, towel or washcloth in 9/35 (26%) videos, water in 7/35 (20%) videos, and wet wipes in 4/35 (11%) videos. Supplies used to protect the skin included a barrier ring in 17/35 (49%) videos, skin barrier in the form of wipe, spray, or cream in 15/35 (43%) videos, and ostomy paste in 6/35 (17%) videos. Supplies used to treat peristomal skin disorders included stoma powder in 12/35 (34%) videos and skin treatments (eg, corticosteroids, and antifungal or other anti-inflammatory medications) in 8/35 (23%) videos. Supplies used to aid adhesion between the pouch and peristomal skin included extenders in 5/35 (14%) videos and belts in 4/35 (11%) videos. Supplies used to prevent or minimize odor issues were odor eliminators in 9/35 (26%) videos and lubricating deodorant in 5/35 (14%) videos. Other general ostomy supplies that were used included plastic bags in 19/35 (54%) to discard the ostomy appliance before disposal, scissors in 16/35 (46%) videos to cut a baseplate to match the size of the stoma, some kind of bag in 4/35 (11%) videos to carry ostomy supplies, and some type of container in 4/35 (11%) videos to rinse the pouch with water.

**Table 6:** Supplies used or discussed in the 35 videos depicting full or partial ostomy appliance change procedures organized by supply category.

Supply category and supply		Videos^a^, n (%)
**Pouching system**		
	2-piece appliance	25 (71)
	1-piece appliance	9 (26)
	Not reported	1 (3)
**Stoma or peristomal skin cleaning**		
	Paper towel or toilet paper	19 (54)
	Adhesive remover wipes	17 (49)
	Adhesive remover spray	14 (40)
	Dry wipes	11 (31)
	Towel or washcloth	9 (26)
	Water	7 (20)
	Wet wipes	4 (11)
	Gauze	2 (6)
	Skin cleanser	2 (6)
	Soap	2 (6)
	Saline	1 (3)
**Peristomal skin protection**		
	Barrier ring	17 (49)
	Skin barrier (wipe, spray, or cream)	15 (43)
	Ostomy paste	6 (17)
	Flow assist device	2 (6)
**Peristomal skin treatment**		
	Stoma powder	12 (34)
	Skin treatments	8 (23)
**Pouch adhesion support**		
	Extenders	5 (14)
	Belt	4 (11)
	Skin adhesive	3 (9)
	Equalizer	2 (6)
	Heating pad	2 (6)
**Odor elimination**		
	Odor eliminator (deodorant or air freshener)	9 (26)
	Lubricating deodorant	5 (14)
	Lubricant	2 (6)
**General**		
	Plastic bags	19 (54)
	Scissors	16 (46)
	Supply bag or bag or ziplock	4 (11)
	Plastic container or water bottle or cup	4 (11)
	Marker	3 (9)
	Size template	3 (9)
	Disposable pad	1 (3)
	Q-tip	1 (3)
	Shower stopper	1 (3)
	Syringe	1 (3)

^a^Calculated as x/35×100%.

#### Underlying Condition and Type of Ostomy

One-third of the narrators who have undergone ostomies (9/28, 32%) did not disclose their underlying condition. Crohn disease was the most frequently reported underlying condition for 10/28 (36%) narrators who have undergone ostomies followed by ulcerative colitis (3/28, 11%), cancer (3/28, 11%), and unspecified inflammatory bowel disease (1/28). Further, 2 (7%) narrators who have undergone ostomies reported other underlying conditions for their ostomy surgery. Per the type of ostomy, ileostomies accounted for 79% (22/28) and colostomies accounted for 14% (4/28). Furthermore, 2 narrators who have undergone ostomies did not report their type of ostomy and no urostomy videos met inclusion and exclusion criteria for final review.

#### Appliance Change Frequency

More than half of narrators who have undergone ostomies (16/28, 57%) did not discuss appliance change frequency. For the remaining narrators who have undergone ostomies (12/28, 43%), there was considerable variability in ostomy appliance change frequency. In general, a majority of narrators who have undergone ostomies (n=10) reported changing their appliance within a 2-5-day interval, 2 every 7 and 15 days, and 1 when the pouch is full. Further, 1 narrator reported various changing frequencies across 4 videos (2-3 days, 3-4 days, every 4 days, and 4-5 days). Additionally, 2 narrators who have undergone ostomies commented on changing the appliance more frequently when having peristomal skin issues (every day or every other day) and emptying the ostomy appliance 5 to 8 times a day.

#### Common Challenges

About one-third of the content creators noted specific issues with supplies or processes that required workarounds or techniques to be implemented. Major challenges included peristomal skin complications (21/65 videos, 32%), leaks around the adhesive area (20/65 videos, 31%), inadequate appliance adhesion (13/65 videos, 20%), and no control over stoma effluent discharge making it difficult to keep the stoma and peristomal skin clean while changing the ostomy appliance (13/65 videos, 20%). Within the videos, the narrators who have undergone ostomies reported peristomal skin complications related to skin irritation and redness due to the output leakage, peristomal skin disorders such as granulomas, or allergic reactions toward specific ostomy supplies and these complications subsequently compromised appliance adhesion, caused pain, and led to further leaks.

Narrators who have undergone ostomies also expressed difficulties related to supplies in 17/65 (26%) videos. For example, supplies leaving residues on the skin compromising appliance adhesion, supplies containing alcohol causing a burning sensation on the wounded skin, variability in supplies’ names causing confusion, and supplies not delivered on time or not working properly for a given individual. Challenges with the design of the ostomy appliance were noted, including the clip of the pouch being uncomfortable, difficulty with cleaning the end of the pouch after emptying, moisture absorption by the barrier ring leading to inadequate adhesion, or a 2-piece appliance being more difficult to apply. Finally, pouch pancaking with the output being trapped at the top of the pouch, pouch ballooning due to the gas accumulating in the ostomy appliance, cutting the baseplate to fit the stoma size, emptying an ostomy appliance and its cleaning, lifestyle adjustments (clothing, diet, relationships, intimacy, travelling, etc), and odor were additional challenges discussed in the videos.

#### Expert Tips and Tricks

The narrators who have undergone ostomies identified various strategies to address the challenges encountered. To overcome peristomal skin complications, narrators who have undergone ostomies (1) emphasized the importance of cleaning peristomal skin (eg, shower without an appliance and using adhesive remover to remove any output residue which could further irritate the skin); (2) avoided supplies containing alcohol to minimize burning sensations on the skin; (3) used a crusting technique which involves the application of stoma powder over sore skin followed by moistening the powder with skin barrier (wipe or spray), letting the powder dry, and repeating the process multiple times [16]; (4) used ostomy paste, or barrier ring to protect and allow healing of the wounded skin by creating a protective barrier between the wounded skin and appliance, or implemented more aggressive skin treatment products such as steroids or silver nitrate based on the consultation with ostomy care nurse specialists; and (5) recommended balance between changing an appliance too frequently or too rarely.

The tips of narrators who have undergone ostomies to avoid leakage included (1) properly fitting the appliance baseplate with the sizing of the opening for the stoma not to be too tight or too big; (2) using a barrier ring or ostomy paste to fill any abdominal gaps and divots; (3) using an ostomy belt, extenders, or medical tape to support the weight of the pouch; (4) trying different supplies (eg, convex pouches for retracted stomas) and different suppliers to identify supplies working properly for a given individual, while also emphasizing not using too many supplies; and (5) emptying an appliance regularly.

Narrators who have undergone ostomies believed that appliance adhesion was improved by (1) cleaning the skin properly using an adhesive remover followed by cleaning with water or some type of wipe to avoid leaving any residues that could compromise adhesion, (2) ensuring the peristomal skin is dry before applying an appliance, (3) wearing an ostomy belt, (4) using an equalizer ring that applies an equal pressure around the stoma or ostomy paste that fills any divots or gaps between appliance and abdomen, (5) applying an appliance on a flat abdominal surface (eg, leaning backward or standing), (6) massaging the baseplate and running a finger around the stoma, and (7) using heat to stimulate baseplate adhesion to the abdomen (eg, hairdryer on low setting, heating pad, or holding hand over pouch). Narrators who have undergone ostomies also acknowledged there is not only one proper way of performing ostomy self-care and that what works for them may not work for someone else. Thus, they recommended trying different supplies and self-care strategies and consulting with ostomy nursing care specialists about supplies selection and their use for skin care.

Concerning managing a frequent and uncontrollable stoma output, some narrators who have undergone ostomies found it effective to eat marshmallows or to fast before changing an appliance to slow down the output, covering the stoma with some type of wipe, paper towel, or gauze while changing the appliance to protect the skin and make it easier to clean, and organizing or preparing all supplies and emptying the pouch before starting to change the appliance.

Narrators who have undergone ostomies conveyed strategies to prevent “pancaking” such as using a lubricant or some type of oil inside the pouch, emptying and rinsing the pouch with water, disabling an appliance filter, and blowing air into the pouch. Further, they used deodorants and “burped” (let the air out of the pouch) their appliance to prevent ballooning. They used an ostomy cover and deodorizing drops along with eating naturally deodorizing food such as kefir, yoghurt, or parsley to deal with the odor. To overcome the challenge of cutting a baseplate to the proper size, narrators who have undergone ostomies traced the stoma size with a marker on the template and saved the template with a marking for repeated use, or ordered the appliance precut from suppliers once their stoma size remained constant. Strategies used to adjust to the postostomy lifestyle included (1) finding and joining a support group, (2) talking to people who “get it,” (3) learning about their stomas (eg, diversity of supplies, proper diet and hydration, or clothing tips), (4) exercising to reduce anxiety, and (5) developing an ability to perform ostomy self-care while also having someone else who can change their appliance.

## Discussion

### Principal Findings

This study uses a novel approach for gaining insight into ostomy care end user (person who has undergone an ostomy) experience and usability with medical device (ostomy appliance change) technology through the identification of strategies and supplies used, and common challenges and needs experienced by people who have undergone ostomies while changing the ostomy appliance and performing self-care. The needs, supply choices, and self-care strategies that people who have undergone ostomies used and discussed reflected the need to address a common challenge of peristomal skin complications following an ostomy surgery [21-23] and demonstrated characteristics of an expert understanding [24-27].

### YouTube as Ostomy Self-Care Resource

This study examined YouTube videos related to the interaction of people who have undergone ostomies with medical device technology and self-care that were provided by and for the public, and the need for additional information related to long-term ostomy self-care in home (nonhospital) settings. In our study, we initially identified 960 ostomy-related videos and analyzed 65 videos with a narrator who has undergone an ostomy that met our inclusion criteria. Each of these videos was viewed 269,000 times on average, liked 3000 times on average, and posted by 28 unique content creators representing a broad range of ages and both, female and male genders. Our study supports current research suggesting that individuals are increasingly seeking health-related information on the web and patients use web-based resources to make health-related decisions and to manage chronic conditions [1-3,35,36]. Patient education and engagement in ostomy self-care are essential for performing self-care tasks and successful management of their chronic condition [18-20,35]. Nevertheless, research evidence shows the pre- and postoperative education provided to people who have undergone ostomies is insufficient [22-25] and new people who have undergone ostomies may not be prepared to gain new knowledge, skills, and attitudes necessary for self-care management of their ostomy immediately post surgery [26,27]. Therefore, our study suggests patients are looking for additional support and resources for ostomy self-care.

The video metadata and data of narrators who have undergone ostomies show that people who have undergone ostomies of various ages and genders are willing to share their personal experiences with ostomy self-care and appliance change procedures on the web and that these videos are viewed by others. Through YouTube, people who have undergone ostomies can share their stories, empower and help other people who have undergone ostomies, and develop a sense of community with other people who have undergone ostomies [10,11], which goes beyond the ability of health care providers not living with a stoma. In our study, narrators who have undergone ostomies created videos depicting ostomy self-care not to substitute health care professional guidelines and recommended practices, but rather to share a lived experience with a chronic illness, and their challenges and wins. They also commented on being able to develop a sense of community, learn from the lived experiences of other people who have undergone ostomies, and adjust to postostomy surgery lifestyle changes such as by finding and joining a support group or talking to people who “get it.” Many of the narrators who have undergone ostomies made disclosures that they were not giving medical advice and commented on positive experiences with and encouraged interaction with ostomy care nurse specialists. The ostomy self-care-related videos depicted what it is like living life with a stoma, and what self-care strategies were found to be effective and what were not. This type of data would be difficult to gain using traditional observational techniques and surveys.

### End User Experience With Ostomy Medical Device Technology

The narrators who have undergone ostomies used a variety of supplies at different frequencies to change their ostomy appliance and discussed challenges related to supplies, challenges with peristomal skin health, appliance adhesion, leakage, and not having control over the stoma output which reflects their user needs. For example, peristomal skin complications were identified by narrators who have undergone ostomies in approximately one-third of analyzed ostomy self-care YouTube videos, and stoma powder (used to treat skin complications) and skin treatments were included and discussed in 12 (34%) and 8 (23%) of the videos depicting an ostomy appliance change procedures, respectively. This finding aligns with extensive research on peristomal skin complications following an ostomy surgery representing a common challenge [30,37,38] and negatively impacting the quality of life of people who have undergone ostomies [39]. The use of peristomal skin protection supplies such as barrier rings, skin barriers, and ostomy paste in 17 (49%), 15 (43%), and 6 (17%) of videos, respectively, and adhesion supplies such as extenders, ostomy belts, and skin adhesives in 5 (14%), 4 (11%), and 3 (9%) of the videos, respectively, manifests the need for properly fitting and adhering external pouching system [17,40]. Thus, the selection of supplies may have been associated with attempts to address appliance adhesion and leakage issues and thus peristomal skin complications.

Narrators who have undergone ostomies reported usability issues with supplies, such as the difficulty with out-of-package baseplate not adhering to “warm” body temperature skin, supplies that are effective in cleaning but compromise peristomal skin health and ostomy appliance adhesion and cause pain (eg, alcohol-based ostomy paste), which call for design improvements of ostomy supplies or redesign related to their properties (eg, baseplate adhesion to peristomal skin, or alcohol-free supplies).

Moldable technology is believed to improve peristomal skin health, reduce the incidence of irritant dermatitis, and be well-perceived by people who have undergone ostomies for its ease of use, ease of learning, and comfort [41,42]. However, in our study, only 3 (9%) of videos depicting the full or part of the ostomy appliance change procedure included a moldable baseplate compared to 25 (71%) of videos where the baseplate was cut to fit or precut and people who have undergone ostomies reported challenges with cutting the baseplate to size. If the stoma hole is too small, the stoma can be damaged, if the stoma hole is too big, the output is more likely to leak on the peristomal skin which in turn will lead to peristomal skin complications. Generally, there are 3 designs of baseplate, precut (cut by supplier), cut-to-size (cut by a person who has undergone an ostomy), and moldable. Moldable baseplate technology does not require cutting the baseplate with scissors but rather using fingers to roll flexible material to size and around (“turtle necking”) the stoma and allows for adjusting the stoma hole to fit snuggly around the stoma while also creating a protective barrier and preventing output from reaching peristomal skin [41]. The narrators who have undergone ostomies did not elaborate on the reason for cutting versus molding baseplate to size, except for 1 video where the narrator who has undergone an ostomy had her husband change the appliance and commented on the molding being “easier to use at first.” Therefore, further studies should be conducted to explain this trend and to more specifically identify user needs. Additionally, future work should determine if people who have undergone ostomies are aware of and educated on the benefits of using moldable baseplate technology versus using cut-to-fit or precut appliances and if moldable appliances are widely available to people who have undergone ostomies.

### Experts’ User-Generated Ostomy Self-Care Strategies

The tips and tricks discussed by the narrators who have undergone ostomies in the videos we examined provide evidence of problem-solving strategies useful for decision-making and ostomy self-management. Ostomy self-care requires complex daily decision-making to solve ostomy-related challenges such as compromised appliance adhesion, leaks, or peristomal skin complications discussed above. People who have undergone ostomies have to monitor their daily output, diet, and stomal or peristomal skin health; perform ostomy appliance changes; and plan future self-management. In previous studies of professional expertise, experts stood out in their ability to identify problems [43], understand functional relationships [44], and use problem-solving strategies [45].

Lippa et al [46] suggest that in the self-management of chronic conditions, such as diabetes, professional expertise may be useful for aiding decision-making and supporting patient’s strategies. Lippa et al [46] also found that diabetic patients who used functional knowledge and problem-solving strategies reported higher levels of adherence to treatment and glycemic control and that descriptive knowledge about diabetes may not necessarily correlate with effective self-management [46]. In our study, narrators who have undergone ostomies demonstrated their expert understanding while elaborating and showing their self-care strategies and adaptation to ostomy-related challenges. These strategies relate to supplies use and organization, ostomy appliance adhesion, baseplate sizing, maintenance of healthy peristomal skin, pouch pancaking and ballooning, and adjustments to lifestyle changes.

Finally, our study of ostomy self-care and end user experiences with ostomy appliance change technology complements the “Ostomy Life Study,” [47] a global effort to gain knowledge about what is it like to live with a stoma and the user experience with ostomy-related supplies. Our study identifies an efficient alternative to traditional observational and survey methods for obtaining end user data that have implications for addressing patient safety and quality of care issues.

### Limitations

Several limitations should be considered when interpreting the results of this study and guiding the direction of future research. In our sample of videos, there were more ileostomies than colostomies, and there were no urostomies, therefore, our findings did not compare different types of ostomies. Further, the narrators who have undergone ostomies were essentially “performing” and demonstrating for the video, therefore, these videos may not capture the routine procedures people who have undergone ostomies use for self-care. In our data analysis, half of the narrators who have undergone ostomies were within an assumed age of 20-30 years and approximately one-third of narrators who have undergone ostomies were in their middle age. This is likely due to most YouTube users falling between the ages of 18 and 44 years [48].

There were also limitations related to demographics in this study. All videos analyzed were narrated in English, but the global representation is unknown. This may limit the global generalizability of this study. Additionally, we did not examine any race, gender, or ethnicity differences in the experiences of people who have undergone ostomies.

### Future Work

Further, it should examine differences in ostomy appliance change procedure and self-care strategies across all the types of ostomies, more specifically, whether ileostomies are more prone to peristomal skin complications considering ileostomies’ output is frequent, more liquid, and high in digestive enzymes [32]; and the prevalence and decision-making of using a moldable versus cut-to-fit appliance technology among people who have undergone ostomies. Future research should also specifically study younger and older people who have undergone ostomies and their experiences, complement this work in other languages and for individuals with experiences in other health care systems, and examine if there are differences among various populations of people who have undergone ostomies.

We identified 28 individual narrators who have undergone ostomies, and their videos were widely viewed. In addition to counting likes, future research should examine who is viewing these videos, what they are learning from them, and the content of viewer comments and discussions related to the video content. The comments analysis could provide a rich discussion around the strategies and ostomy self-care provided in the ostomy self-care videos.

In our study, we purposely did not include any video content from medical professionals or health care professional organizations because it can contain different information about ostomy self-care strategies than what is practiced by people who have undergone ostomies in home settings [35]. We intended to evaluate end user experiences with ostomy self-care rather than how people engage with YouTube videos created by health care professionals. However, we recognize there is value in comparing user-generated information to what clinicians and health-related government organizations may communicate about ostomy self-care as they use YouTube to disseminate health information [1] that is considered to be the most reliable and accurate information [6]. Future research should examine the content in the ostomy self-care videos created by health care professionals and professional organizations and evaluate how they compare to the user-generated ostomy self-care YouTube videos examined in this study.

### Conclusions

This study used a novel approach for ostomy care (YouTube videos) to gain insights into ostomy self-care in home settings and end user interactions with medical devices while performing self-care, which are difficult to gather using traditional behavioral techniques. Using YouTube or other publicly available content created by individuals dealing with a chronic condition is a low-cost means to identify successful strategies, potential design problems, and potential workaround for issues that would be difficult to obtain in other study designs and approaches. The analysis of 65 videos showed that people who have undergone ostomies are willing to share their personal experience with ostomy self-care on the web and that these videos are viewed by the public. The findings also identify user needs related to medical device technology and indicate that narrators who have undergone ostomies were able to use problem-solving strategies for decision-making and successful ostomy self-management. This study demonstrates the potential value of these videos for filling the gap between health information delivered by health care professionals and the lived experience of individuals dealing with a chronic condition.

Additional information related to long-term ostomy self-care in a home (nonhospital) setting and the differences between what is clinically recommended and how the procedures and supplies are actually used by individuals living with ostomies needs to be more clearly understood for the design and testing of future ostomy supplies as well as for patient education from clinicians. Future research could use these approaches to address other types of clinical information that other clinical populations need to learn or be able to access when at home as well as strategies to design better medical devices for home health care in the future.
